# An Interlude and Two Reminiscences

**Published:** 1918-01-05

**Authors:** 


### AN INTERLUDE AND TWO REMINISCENCES.

By "CADUCEUS."

The later turns of this world drama (for all conflict is dramatic) are depressing you a little? It is time, then, at this holiday season too, for something fresh in this miscellany I am allowed to contribute. No more satirising, no more blowing-up, no more "shop" even just now; but in place of these brow-wrinkling themes, a pair, or let us say, rather, a couple (isn't that the technical term in mechanics for two forces opposed in a certain way?), of little reminiscences which may possibly prove of interest to members of a learned profession and its associates. For their proper narration one ought to have the visual memory of the artist, but as that, for good or ill, is lacking, you must be content with an account instead of a picture.

The first is of a luncheon party at a London hotel to meet a famous novelist. An old man, as well as a great one, he came late, causing thereby in the breast of one of the ladies present offence she evidently felt unequal to avenging. The *susurrus* of introductions may have reached other ears than those of the circle of guests, for it was said that two or three strangers in the big room circumnavigated the table where the celebrity sat in order to get a thorough look at him. "I think," he was reported to have, rather unpatriotically, observed of these matter-of-fact persons, "I could *smell* who they were." Only one feature of his appearance would have struck one, and that was the light-coloured eyes. When I saw them I remembered an intellectual young Boer whose boyish face was undistinguished except for uncommon light-brown eyes, the colour of a lion's; I remembered, too, the mild lightish-blue gaze of a Scotch golf professional, in whom self-confidence, that Atlas that bears up his nation, was, curiously, lacking; and the pleasure born of recognising this physiognomical phenomenon was the first bit of solace for the mental gooseflesh formal converse can bring on. In Henry James the eyes contradicted flatly the rest of him. *That* was a prosperous old banker; *these* were shy and elusive, dreamy and distrustful "like a wild horse's." The set, earnest, studious look he has both in Mr. Sargent's portrait in the old "Yellow Book" and in Mr. Coburn's photograph study seem to me like nature faking. The "bar of Michael Angelo" which these show he no doubt had, but you do not look at a newcomer in profile, you wish to see him full face; moreover, many people utterly free of talent have big frontal sinuses. His manner matched the eyes, not the figure. The thought gave me an unhappy shudder:—with what severity of truthfulness he could have described a manner like his own!

"It was a question—er—of—um—illustrating—a frontispiece, that is—some stuff of mine [more modestly than ever]—'the Golden Bowl' the particular thing was called, and—er—we wanted something—something of a curiosity shop—a photograph of something that came [again lowering and hurrying his tone]—that really came out of my head. And, well, Mr. Coburn and I hunted about London for two days—no good. The third day we found it. It was in a street up there behind [Gray's Inn, I think he said, but am not sure]. Coburn fancied it at once. I—er—thought it likely; but—um—went on a little, leaving him sizing it up outside. Further on I saw the exact thing. I rushed back [then, correcting himself with a remembrance of weight and age, neither of which has spoilt his wonderful talent]—I went back to him. 'Come and see what I've found.' I said. He didn't want to come. 'Have you got something better than this?' 'Come and see the real thing' [this leaning back in the arm-chair with a pleased smile of remembrance and a supply of ease of manner]. So that was how it was, and [sitting forward and preparing to move] it just shows how you can find *anything* in London."

He considered photography a poor art, as, of course, he would. On the other hand, he thought better of Mr. Bernard Shaw than you might have expected. He thought well of Mr. Coburn, but not of his portrait of Henry James. This may have been referable to that little matter about the eyes already touched upon, but is much more likely to be the altogether general human trait of not thinking one's likeness good-looking or distinguished enough. We all row in that galley.

. . . . . .

I prepared you for an account instead of a picture, and so far have been as bad as my word. But this second reminiscence may have something pictorial about it, for it pertains to childhood, and children are supposed to remember in pictures, like artists and savages. Have you read George Moore's "Ave"? You get it doubly there. A small boy, then, went to a school which a big boy had recently left. At the end of a long, wearisome stretch of existence, the whole unlucky thirteen weeks of a term, there came a blessed holiday morning, and on the drab desks appeared the whitey-pinky-creamy school magazine, clean and fresh from the printer. There were the names of those sitting around mysteriously apotheosised in print. There, too, was a new woodcut, the feature of this number, an initial "E" to the *Entre Nous* column, a comic newsboy leaning over the middle limb of the letter, the school mag. in his hand and under his arm. That, they said, was an important and not inexpensive addition since the time of the big boy aforesaid, the former editor. And who was he? A vision of an earlier number comes back to me; on the outside, "The So-and-So School Magazine," and under it, in large letters right across the page:—

"Edited by Alfred C. Harmsworth."

Inside there were a dozen or so lines of verse—later reflection suggests, of course, a sonnet—upon the subject of Hampstead Heath. Mostly, I

remember, they were signed by the editor. Never, the head of the school has said since, was boy more father to the man than this editor. What would benefit the circulation went in, and the rest stopped out. In subsequent issues mention of the doings of old boys began to include references to successful journalism; I think, looking back, I can visualise the word *Answers*. That is my last little blur of an impression. You remember Cossington and the school magazine in Mr. Wells' masterpiece "The New Machiavelli"? That is true to life, and may well be, for H. G. Wells was science master at this now deceased very preparatory school after the time of which I write. Yes, Mr. Wells' picture is the one to go and look at this very Christmas. Mr. Bennett's "What the Public Wants," unfortunately, you cannot see. Thank you for listening to my table talk.

